# Personality Profile and Clinical Correlates of Patients With Substance Use Disorder With and Without Comorbid Depression Under Treatment

**DOI:** 10.3389/fpsyt.2018.00764

**Published:** 2019-01-11

**Authors:** Julia Elena Marquez-Arrico, Laura Río-Martínez, José Francisco Navarro, Gemma Prat, Ana Adan

**Affiliations:** ^1^Department of Clinical Psychology and Psychobiology, School of Psychology, University of Barcelona, Barcelona, Spain; ^2^Department of Psychobiology, School of Psychology, University of Malaga, Malaga, Spain; ^3^Institute of Neurosciences, University of Barcelona, Barcelona, Spain

**Keywords:** comorbid depression, substance use disorder, personality profile, dual disorders, alternative five factor model

## Abstract

**Background:** Among patients with substance use disorder (SUD), major depressive disorder (MDD) is highly prevalent. Even though, personality plays an important role in treatment outcomes for individuals with SUD and SUD + MDD, few studies have explored personality characteristics in these samples. This study aims to describe personality for patients with SUD taking into account the comorbid MDD, using the Alternative Five Factor Model (AFFM). We also aim to compare SUD + MDD patients with population norms and to elucidate possible personality clinical correlates.

**Methods:** For our study, 116 male patients undergoing for SUD treatment were divided in two groups: SUD only (*N* = 58) and SUD + MDD (*N* = 58). To examine personality, we used the Zuckerman-Kuhlman Personality Questionnaire and multiple analyses of covariance were performed to identify differences. In a first analysis, age was introduced as a covariate whereas in a second analysis the continuous variables that showed to have a discriminant value for the groups were added as covariates. Variables predicting the presence of dual diagnosis and personality clinical correlates were analyzed by logistic and linear regression models, respectively. We observed that patients with SUD + MDD show distinctive personality characteristics compared with patients with SUD only and population norms.

**Results:** According to the AFFM, SUD + MDD patients are characterized by higher Neuroticism-Anxiety (positively associated to depressive symptoms) and Impulsivity; and by lower Parties and Friends. Moreover, the probability of having a dual depressive disorder was represented by the amount of medications and substances used. The preference for hard work and the energy self-reported levels (Work Activity trait) are linked to these clinical variables rather than to the presence/absence of a dual depressive disorder.

**Conclusions:** Even when controlling clinical variables related to a higher probability of having a dual depressive disorder, the Neuroticism-Anxiety is a personality trait that strongly differentiates between SUD only and SUD + MDD patients. Further investigation is needed to explore the role of this personality trait as endophenotype in dual depressive men. Our results underline the importance of a dimensional understanding of personality and its clinical correlates among patients with SUD + MDD; this approach could provide us information on specific treatment strategies to improve the prognosis of patients.

## Introduction

Research has shown than personality characteristics need to be considered in order to accomplish a deeper knowledge which allows to improve both theoretical and practical comprehension of substance use disorders (SUD) (1–3). Moreover, SUD and comorbid major depression disorder (MDD) is highly prevalent (4, 5) and personality characteristics also influence on clinical features and treatment response among patients with both disorders (6, 7). Having a SUD and a comorbid MDD (SUD + MDD) is strongly associated to alcohol/cocaine dependence (8, 9), more severe depressive episodes (10) and major clinical complications during treatment (11, 12) compared with having SUD only. Therefore, the study of clinical features of patients with SUD + MDD is an interesting topic for both researchers and clinical practitioners.

In this sense, as a result of such complications associated to comorbidity of both disorders, recent research has attempted to identify personality variables related to SUD with psychiatric comorbidity and tried to elucidate clinical correlates (13–16). The majority of the research about personality in patients with SUD has been conducted from a psychobiological perspective which has shown to be more sensitive to these patients' specific personality characteristics. Accordingly, studies have found that elevated scores in Impulsivity and Sensation Seeking personality traits are associated to developing a SUD (17), to a stronger craving for patients with SUD, higher severity of addiction (18, 19) and more relapses (17, 20). Moreover, low scores in traits like Reward Dependence, Persistence, Cooperation, and Self-Transcendence (from Cloninger's Temperament and Character Inventory) are strongly related to dropping-out SUD treatment (21).

On the other hand, research about personality in patients with MDD indicated that high Extraversion, Agreeableness, and Conscientiousness (from NEO's Five Factor Inventory) significantly reduced the risk of a new depressive episode, while only Neuroticism predicted a new episode (7).

Regarding personality in patients with SUD + MDD, data up to now have shown that, compared with SUD or MDD only patients, they have higher Neuroticism (from NEO Five Factor Inventory) (22), Novelty Seeking (23, 24), Harm Avoidance (25), and lower Persistence, Self-Directedness, and Cooperativeness (23, 24) (from Cloninger's Temperament and Character Inventory). These findings are relevant as personality is related to the clinical course of patients with and SUD + MDD, with high scores in Harm Avoidance being associated to dysphoric episodes (26), severe depressive symptomatology (27), and poorer response to antidepressant treatment (28).

In sum, the influence of specific personality dimensions on addictive disorders and psychopathology conditions like MDD has accumulated sufficient scientific background to be worth considering (29). However, to our knowledge the possible differences between patients with SUD only and with SUD + MDD have not been studied to analyze the role of depression in personality traits of patients with drug dependence. For all these reasons, we have chosen to explore such issue using the Alternative Five Factor Model of personality due to its theoretical background, psychobiological perspective, cross-cultural validity (30), and good psychometric properties in psychiatric samples (31).

As far as we know, this is the first study that uses the Alternative Five Factor Model to examine personality differences between patients with SUD and SUD + MDD, which also aims to compared them with population norms. Additionally, we seek to identify whether personality characteristics could be associated to clinical features related to both SUD and MDD diagnoses.

## Materials and Methods

### Participants

Our total sample was comprised by 116 male patients (mean = 40.58 years; *SD* = 8.03) undergoing treatment for their SUD in public and private centers, who were divided in two groups regarding their diagnosis: SUD only (*N* = 58) and comorbid SUD + MDD (*N* = 58). All participants were referred to the study by their psychologist and psychiatrist; those providing written informed consent were included in the study and assessed by an experienced postgraduate psychologist. The majority of the measures were collected during the morning because patients from our treatment centers are under therapeutic interventions that are highly structured following the light-dark rhythm. The inclusion criteria were as follows: (1) current diagnosis of SUD, according to DSM-5 (with no depressive symptoms to be included in the SUD only group) (32), in remission for at least 3 months and with no relapses, confirmed by urinalysis in each treatment center; (2) male gender (as this is the most prevalent gender among people with a SUD diagnosis and in patients from our treatment centers); (3) aged 22–55 years; (4) those patients having a co-occurring diagnosis were only included if they met criteria for MDD. The exclusion criteria were: (1) meeting DSM-5 criteria for a current substance-induced psychiatric disorder or a psychiatric disorder due to medical condition; (2) unstable or uncontrolled psychiatric symptomatology; (3) inability to complete study instruments.

The University of Barcelona ethics committee's approved this study in accordance with the ethical standards of the Helsinki declaration. Participants were not economically compensated for their participation but they received a report with their personality profile through the professionals of their respective treatment centers.

### Measures

#### Sociodemographic and Clinical Measures

Current diagnosis of SUD and MDD was referred by treatment providers of each respective patient and confirmed using DSM-5 criteria. For collecting demographic and clinical variables we used the Structural Clinical Interview for DMS-IV-TR Axis I Disorders (SCID-I) (33), as the Spanish version of the SCID from DSM-5 was not available during the developing of our study, along with a clinical interview designed for our study.

The Spanish version (34) of the Drug Abuse Screening Test (DAST-20) (35) was used to measure severity of the SUD in both groups; this instruments provides a total score which ranges from 0 to 20 and reveals the severity of addiction. The total score is understood by the following cutoff points: 1–5 low, 6–10 intermediate, 11–15 substantial, and 16–20 severe (a higher score means a higher severity of addiction).

For measuring depressive symptoms in the SUD+MDD group we used the Hamilton Depression Rating Scale (HDRS) 17-item (36); in this case the cutoff points were: 0–7 no depression, 8–13 mild, 14–18 moderate, 19–22 severe, and ≥23 very severe depression.

#### Personality Assessment

Personality through the Alternative Five Factor Model was assessed using the Spanish version of the Zuckerman-Kuhlman Personality Questionnaire (ZKPQ) (37). This questionnaire is composed by five scales or personality factors. The first scale is Neuroticism-Anxiety (N-Anx); it is formed by 19 items and describes a tendency to negative emotions and sensitivity to criticism. The second scale is Activity (Act), formed by 17 items, which describes the need for general activity and the preference for hard and challenging work. The Act scale has two different subscales: General Activity (GenAct; 9 items) and Work Activity (WorkAct; 8 items). The third scale is Sociability (Sy), composed by 17 items, that explains the preference for having friends and spending time with them. Two subscales can also be obtained from Sy, these are Parties and Friends (Part; 9 items) and Isolation Intolerance (Isol; 8 items). The fourth scale is Impulsive Sensation-Seeking (ImpSS), formed by 19 items, that involves a lack of planning and the tendency to act without thinking and looking for excitement. The ImpSS scale gives two subscales: Impulsivity (Imp; 8 items) and Sensation Seeking (SS; 11 items). The fifth and last personality scale is Aggression-Hostility (Agg-Hos), composed by 17 items, which explain the tendency to express verbal aggression as well as being rude to others. Finally, the ZKPQ includes also an Infrequency scale (Infreq) formed by 10 items that is used to detect inattention to the task or understood as a validity measure rather than a normative scale.

### Statistical Analyses

The main descriptive data (means, SD, frequencies) were calculated for all variables measured for both SUD and SUD+MDD groups. Differences in sociodemographic and clinical variables were explored with ANOVA or Chi-square (χ2) test for continuous and categorical variables, respectively. To explore which sociodemographic and clinical variables could be related to a greater probability of being in the dual disorder group (SUD+MDD) we carried out logistic regression analyses through stepwise regression method. Logistic regression coefficients and their standard errors were exponentiated to create odds-ratios (ORs) and their 95% confidence intervals. Continuous predictors were divided into categories to minimize the effects of extreme values to stabilize associations.

Intergroup differences for the ZKPQ scales, considering the SUD and SUD+MDD diagnoses, were examined by multivariate analyses of covariance (MANCOVA) introducing group as an independent variable and age as a covariate since it could be a confounding factor (38). In addition, a second MANCOVA analysis was performed adding as covariates the continuous variables pointed out in the logistic regression analysis as discriminating factors between the groups. In both cases we performed one MANCOVA for the scales and another one for the subscales. *Post-hoc* analyses were Bonferroni corrected and we estimated partial Eta-square (η_*p*_^2^) to measure the effect size. Cronbach's alpha coefficient of internal consistency was calculated for the ZKPQ scales; as well as T scores, according to the Spanish population norms (37), for the scales and subscales.

Finally, to identify the possible relationships among the ZKPQ personality scales and clinical variables (SUD and MDD related) bivariate correlation analyses were performed. Only the scales showing significant associations with clinical variables were entered in the subsequent multiple linear stepwise regression analysis. All data were analyzed using the SPSS/PC software, version 24.0, and all statistic tests were bilateral with a *p* ≤ 0.05.

## Results

### Participant's Characteristics

The main sociodemographic and clinical features of the sample are described in Table 1. Concerning sociodemographic variables, groups were different in marital status (*p* = 0.036) and economic situation (*p* = 0.008). Patients with SUD + MDD were more likely to be single or separated/divorced than patients with SUD only, who were more likely to be married or with a stable partner. In addition, while patients with SUD + MDD were characterized by being unemployed or having a disability pension, patients with SUD only were working or having a sick leave due to SUD treatment.

**Table 1:** Sociodemographic and clinical data.

	**SUD + MDD**	**SUD only**	**Statistical contrast**
**SOCIODEMOGRAPHIC DATA**			
Age (years)	42.05 ± 6.85	39.10 ± 9.36	*t*_(98)_ = 1.94
Marital status			*χ^2^*_(4)_ = 10.28^*^
Single	44.8%	37.9%	
Stable partner	3.4%	17.2%	
Married	10.3%	19%	
Separated/divorced	37.9%	20.7%	
Widower	3.4%	5.2%	
Years of schooling	10.29 ± 2.68	10.84 ± 2.74	*t*_(98)_ = 1.10
Economic situation			*χ^2^*_(4)_ = 13.74^**^
Active	8.6%	22.4%	
Unemployed	39.7%	32.8%	
Disability pension	31%	8.6%	
Sick leave (due SUD treatment)	12.1%	20.5%	
No income	8.6%	15.7%	
**CLINICAL DATA**			
Medical disease comorbidity	50%	31%	*χ^2^*_(5)_ = 4.32^*^
Hypercholesterolemia	12.1%	0%	
Respiratory system disease	12.1%	8.6%	
Hepatitis	12.1%	6.9%	
Diabetes	6.9%	3.4%	
HIV	3.4%	1.7%	
Other	3.4%	10.3%	
Daily number of medications	2.05 ± 1.41	0.55 ± 1.05	*t*_(114)_ = 6.48^***^
Type of medication prescribed			
Antidepressants	74.1%	20.7%	*χ^2^*_(1)_ = 33.23^***^
Anxiolytics	53.4%	6.9%	*χ^2^*_(1)_ = 28.83^***^
Mood stabilizers	21.1%	8.6%	*χ^2^*_(1)_ = 5.10^*^
Interdictor	10.3%	10.3%	*χ^2^*_(1)_ = 0.01
Other	11.9%	20.7%	*χ^2^*_(1)_ = 25.14^*^
Quantity of substance used^a^	2.83 ± 1.46	1.97 ± 0.86	*t*_(114)_ = 3.87^***^
Alcohol	84.5%	75.9%	*χ^2^*_(1)_ = 1.36
Cocaine	79.3%	74.1%	*χ^2^*_(1)_ = 0.24
Cannabis	44.8%	32.8%	*χ^2^*_(1)_ = 2.60
Hallucinogens	20.7%	6.9%	*χ^2^*_(1)_ = 4.64^*^
Opioids	27.6%	5.2%	*χ^2^*_(1)_ = 10.63^***^
Sedatives	15.5%	1.7%	*χ^2^*_(1)_ = 7.04^**^
DAST-20	14.29 ± 3.14	13.64 ± 3.76	*t_(_*_114)_ = 0.10
History of suicide attempts	41.4%	17.2%	*χ^2^*_(1)_ 8.16^**^
Number of lifetime suicidal attempts	0.76 ± 1.15	0.22 ± 0.56	*t*_(114)_ = 3.19^**^
Mean abstinence period (months)	6.97 ± 4.50	8.84 ± 6.16	*t*_(114)_ = 1.88
Substance use disorder age onset (years)	18.88 ± 7.11	19.53 ± 5.90	*t*_(114)_ = 0.54
History of substance use (years)	23.17 ± 9.91	19.57 ±10.31	*t*_(114)_ = 1.91
Major depressive disorder age onset (years)	30.81 ± 8.72		
HDRS	11.26 ± 4.81		

Means and standard deviation or percentages, and statistical contrasts.

SUD + MDD, substance use disorder with comorbid depression; SUD, substance use disorder; DAST-20, drug screening test; HDRS, Hamilton Depression Rating Scale.

a Percentages will not equal 100 as each participant may take more than one substance.

* p < 0.05;

** p < 0.01;

*** *p ≤ 0.001*.

On the other hand, analyses for clinical variables revealed that groups differed in the presence of medical disease comorbidity (*p* = 0.038), daily number of medications (*p* < 0.001), quantity and type of substances used (*p* < 0.001), history of suicide attempts (p = 0.004) and number of lifetime suicidal attempts (*p* = 0.002). In this sense, SUD+MDD patients had a higher rate of medical disease comorbidity (such as hypercholesterolemia, respiratory system diseases, or hepatitis) and used a higher daily amount of medications (antidepressants, anxiolytics and mood stabilizers) per day compared to SUD only. Some of the patients with SUD only were using antidepressants, but in all cases these medications were prescribed for sedative or anxiety reasons, as well as, for managing addiction. Moreover, patients with SUD + MDD showed a higher quantity of substances used and they were more likely to use hallucinogens, opioids, and sedatives than patients with SUD only. Finally, we also observed that a history of suicide attempts and number of lifetime attempts was more probable in patients with SUD+MDD than in those with SUD only. No other differences were found.

Regarding clinical measures, we observed that both groups presented substantial severity of addiction according to the cut off points of DAST-20. Patients with SUD + MDD had mild depressive symptomatology and only 8.6% (*n* = 5) were asymptomatic at the time of participating in our study according to HDRS scoring.

The logistics regression analysis with sociodemographic and clinical variables as predictors for being in the dual disorder group (SUD + MDD) showed that only the model with the daily number of medications (β = 0.93; OR 2.55; 95% CI 1.73–3.75; *p* < 0.001) and the quantity of substances used (β = 69; OR 2.01; 95% CI 1.30–3.78; *p* = 0.002) correctly predicted the patient's group for the 76.5% of the subjects (Nagelkerke's R^2^ = 0.44).

### Results in Personality

According to Cronbach' alpha coefficients for the five ZKPQ scales, internal reliability was appropriated for the total sample with the following results: N-Anx 0.846, Act 0.746, Sy 0.773, ImpSS 0.850, and Agg-Host 0.729.

MANCOVA analyses for the five ZKPQ scales revealed differences among the groups for N-Anx and Sy. The subsequent MANCOVA analyses for the subscales indicated differences for WorkAct, Part, and Imp (see Table 2).

**Table 2:** Descriptive statistics (mean and standard error) for the Alternative Five Factor Model of personality and MANCOVA results with *F* and eta square ($\eta_{\text{p}}^{2}$) tests according to the diagnosis and considering different covariates.

			**Age as a covariate**		**Age, quantity of substances used, and daily number of medications as covariates**	
**Personality traits**	**SUD + MDD**	**SUD only**	***F***	**$\eta_{\text{p}}^{2}$**	***F***	**$\eta_{\text{p}}^{2}$**
Neuroticism-anxiety	11.09 ± 0.50	7.48 ± 0.45	20.66^***^	0.155	12.89^***^	0.104
Activity	7.84 ± 0.30	8.91 ± 0.32	2.50	0.022	0.02	0.001
General activity	4.41 ± 0.30	4.67 ± 0.18	0.32	0.005	0.36	0.003
Work activity	3.61 ± 0.23	4.56 ± 0.18	10.24^**^	0.083	3.04	0.027
Sociability	5.72 ± 0.49	7.07 ± 0.45	4.42^*^;	0.038	1.14	0.010
Parties and friends	2.43 ± 0.26	3.29 ± 0.26	5.65^*^;	0.048	3.98^*^;	0.035
Intolerance to isolate	3.29 ± 0.30	3.78 ± 0.30	1.38	0.012	0.01	0.001
Impulsive sensation-seeking	9.51 ± 0.55	8.63 ± 0.54	1.26	0.011	1.61	0.014
Impulsivity	4.19 ± 0.31	3.16 ± 0.35	6.10^*^;	0.051	4.37^*^;	0.038
Sensation seeking	5.26 ± 0.41	5.47 ± 0.35	0.15	0.001	0.01	0.001
Aggression-hostility	7.96 ± 0.37	8.38 ± 0.40	0.58	0.005	0.69	0.006

SUD + MDD, substance use disorder with comorbid Major Depression Disorder; SUD, substance use disorder.

* p < 0.05;

** p < 0.01;

*** *p ≤ 0.001*.

For the SUD + MDD group, we observed higher N-Anx (*p* ≤ 0.001) and lower Sy (*p* = 0.038) than for SUD only. Moreover, also for the SUD + MDD group, we found lower WorkAct (*p* = 0.002)and Part (*p* = 0.019), and higher Imp (*p* = 0.015) compared to SUD only.

On the other hand, the second MANCOVA analysis considering age, the daily number of medications, and the quantity of substances used as covariates eliminated the differences in WorkAct and Sy appreciated previously (see Table 2). However, the differences observed for the personality traits of N-Anx, Part, and Imp are still maintained in the second analysis, but with lower statistical power. In this sense, patients with SUD+MDD presented a higher N-Anx (*p* ≤ 0.001), lower Part (*p* = 0.049), and higher Imp (*p* = 0.039) than patients with SUD only.

**Figure 1:**
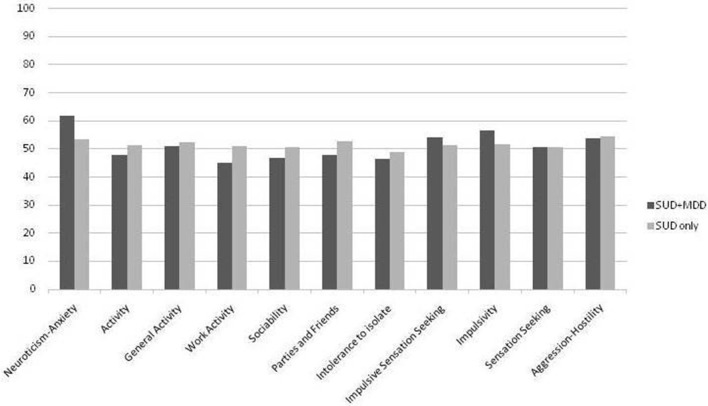
T scores according to population norms for the two groups in the scales and subscales of the ZKPQ questionnaire. SUD + MDD, Substance Use Disorder and comorbid Major Depression Disorder. SUD, Substance Use Disorder.

Finally, the calculation of the T scores (mean = 50; *SD* = 10) according to population norms for the ZKPQ scales and subscales indicated that N-Anx was above average for both groups but this was more remarkable (+1 *SD*) for patients with SUD+MDD (see Figure 1). In addition, only for patients with SUD + MDD, WorkAct, and Sy were both below average while Imp was higher than population norms.

### Clinical Correlates of Personality

Stepwise regression analyses for the ZKPQ scales showed that only for patients with SUD + MDD personality was linked to clinical variables, either SUD or MDD related. We observed that for patients with SUD + MDD depressive symptoms (HDRS) scoring was positively associated to N-Anx (β = 0.365; *R*^2^ = 0.106; *t* = 2.215; *p* = 0.034); the age of SUD onset was positively linked to Act (β = 0.290; *R*^2^ = 0.068; *t* = 2.270; *p* = 0.027); and severity of addiction (DAST-20 scoring) was positively related to Agg-Hos (β = −0.309; *R*^2^ = 0.079; *t* = 2.388; *p* = 0.020). We did not find any more significant correlates of personality among other variables.

## Discussion

Our study sought to explore personality differences between patients with SUD taking into account the presence of a comorbid MDD using the psychobiological approaching of the Alternative Five Factor Model of personality. We also aimed to compare data from our sample with population norms, as well as to identify clinical correlates of personality dimensions considering SUD and MDD comorbidity.

In the first place, sociodemographic and clinical results are in line to previous data regarding patients with SUD + MDD and SUD only (8, 9). Moreover, extending previous findings (1, 12, 23), we observed differences between the groups which suggest that patients with SUD + MDD present a sociodemographic and clinical profile characterized by variables that are associated with a worse prognosis and treatment outcomes (e.g., being single/separated, disability pension/unemployment, medical disease comorbidity, suicide attempts).

Regarding sociodemographic and clinical variables that could predict whether or not the patient is in the dual disorder group (SUD + MDD) we observed that from all considered variables only the daily number of medications and the quantity of substances used were involved. Thus, the higher daily amount of medication and substances used implies a higher probability of being a dual depressive patient. Such observations are in line with previous studies since SUD + MDD are linked to more physical illnesses (39), and psychiatric symptoms (40), as well as polydrug use (8, 18). These observations should be considered more in depth by future studies about personality in SUD + MDD patients.

In the second place, our findings for personality characteristics pointed out that patients with SUD + MDD were more likely to be emotionally upset, tense, worried, fearful, indecisive, lack of self-confidence, and sensitive to criticism (higher N-Anx), than patients with SUD only and population norms. These results, which are observed even when controlling the quantity of substances used and the daily number of medications, are consistent to previous data (13) and suggest a possible additional difficulty for patients with comorbid MDD to regulate their emotions (41) and overcome their addiction (42); since it is a personality trait formed by characteristics that are associated to a non-adequate treatment adherence, more relapses, and worse treatment outcomes (11, 12, 22). Furthermore, the N-Anx personality trait was a clinical correlate of depressive symptomatology, which is in line with previous studies identifying Neuroticism as a predictor of recurrent depressive episodes (7).

In addition, when only age is considered as a covariate patients with SUD+MDD, show a lower preference for hard and challenging work, an active busy life or high-energy levels (lower WorkAct) compared with patients with SUD only and population norms. Even though these observations are coherent with previous findings and with the presence of depressive symptoms, such as tiredness and low energy levels (11, 32), this difference between the groups is no longer observed when controlling the effect of drugs used and medications per day. Therefore, preferring hard and challenging work and self-reported energy levels may to be conditioned by the quantity of substances used and medications per day rather than associated to the presence/absence of a dual depressive disorder. These observations should be considered by future studies and when designing treatments for addiction, since to make the most of the interventions, physical exercise may be included as it has proven evident benefits (43, 44). Moreover, the age of SUD onset for patients with SUD + MDD was a clinical correlate for the personality trait of Act. In this sense, those patients with an older age of SUD onset showed a higher preference for being active, which it could be possible explained by substance use limiting the span of activities done as a result of prioritizing addiction related behaviors (15). On the other hand, the activity scale of ZKPQ questionnaire appears to be sensitive to circadian typology (45, 46), and the SUD treatment imposes a morning circadian functioning (39, 47) which could influence energy levels of patients during treatment. Future studies should analyze the role of this individual difference in addict and dual disorder patients with measures before, during, and after treatment.

Furthermore, consistent with previous work (13) and even when controlling the effect of drug use and daily number of medications, we also appreciated that patients with SUD + MDD were characterized by a preference for being alone instead of spending time with family and friends (lower Part) compared to SUD only and population norms. The difference observed in the first place for Sy is no longer founded once drug use and medications are controlled, hence, these two variables could be influencing the social needs in patients of our sample. Such covariates should be considered by future studies about personality in patients with a dual depressive disorder. Overall, our observations could represent a higher difficulty for patients with SUD + MDD since social support has positive implications for recovery in drug dependence patients (48) as well as for preventing recurrent depressive episodes (7).

Finally, we observed that patients with SUD + MDD presented a higher tendency to act impulsively without thinking (higher Imp) than patients with SUD only and population norms. These findings are in line with previous data, are also observed once the effects of the quantity of drug use and medications are controlled, and suggest that, even though impulsivity is a personality trait associated with substance abuse, when comparing patients with SUD regarding their MDD comorbidity, those with SUD + MDD tend to show a stronger presence of impulsivity. Hence, having both disorders could be indicating a greater vulnerability for relapses or poor treatment outcomes (1, 49).

Overall, to our knowledge this is the first study that explores personality traits according to the Alternative Five Factor Model in patients with SUD taking into account the presence of a comorbid MDD. Even though further studies are needed, our research could be considered a relevant approach to inform professionals to prioritize aims during therapeutic interventions for patients with SUD + MDD, since they exhibit different personality characteristics than patients with SUD only. For example, interventions for patients with SUD + MDD could emphasize emotion and impulsivity management, as well as strategies for extending their social network.

On the other hand, this study has limitations that should be considered. As we used a cross-sectional design we could not determine the manner in which diagnosis (SUD and SUD + MDD), sociodemographic, and clinical variables interact with personality dimensions. In this sense, longitudinal and larger studies with other statistical methods are needed to allow more robust approaches to assess such interactions. Moreover, we only included male patients in our sample, with a large age range and differing in the daily number of medications used depending on their group. In this sense, it is widely known that dual disorder patients had more clinical complications and tend to use more medications than patients with SUD only related to second diagnosis, but future research is needed to clarify the role of these factors among personality traits that could be conditioned by pharmacological treatment (e.g., activity and sociability). All of these limitations together with the moderated effect sizes of our results may limit the generalizability of our findings.

## Conclusions

This work could be considered a first approach for understanding personality characteristics in a sample of patients with SUD taking into account MDD comorbidity, from the psychobiological perspective of the Alternative Five Factor Model of personality. Our findings suggest that, compared with patients with SUD only and population norms, patients with SUD + MDD show distinctive personality characteristics. According to the Alternative Five Factor Model of personality, patients with SUD+MDD are characterized by higher N-Anx (positively associated to depressive symptoms) and Imp; as well as by lower Part compared to patients with SUD only. In this line, our results pointed out that the personality traits of N-Anx and Imp which were previously associated to drug use, in our sample are traits linked to the presence of comorbidity. The strongest difference identified between patients with SUD + MDD and SUD only is founded for the N-Anx trait. Hence, our findings are suggesting that further investigation is needed to explore the possible role of N-Anx as an endophenotype for a dual depressive disorder. Moreover, the probability of having a dual depressive disorder was represented by the amount of medications and substances used; and the tendency for preferring challenging work, as well as self-reported energy levels, is conditioned by these two variables rather than by the presence of comorbid depression in patients with SUD. Our findings are relevant since previous research has linked similar characteristics to worse prognosis and major clinical difficulties and observations may be considered during therapeutic interventions for patients with SUD + MDD.

## Author Contributions

AA conceived the original idea for the study, sought funding, and wrote the protocol. JM-A collected the sample data and carried out all the data analyses with input from AA. JM-A and AA wrote the manuscript with input from LR-M, JN, and GP. All authors have approved the final manuscript.

### Conflict of Interest Statement

The authors declare that the research was conducted in the absence of any commercial or financial relationships that could be construed as a potential conflict of interest.
